# Looking inside an injection system

**DOI:** 10.7554/eLife.50815

**Published:** 2019-09-17

**Authors:** Sophie A Howard, Alain Filloux

**Affiliations:** MRC Centre for Molecular Bacteriology and Infection, Department of Life SciencesImperial College LondonLondonUnited Kingdom

**Keywords:** contractile injection system, metamorphosis, *Photorhabdus*, toxins, T6SS, *E. coli*, Other

## Abstract

The proteins injected by bacteria into eukaryotic organisms can lead to fates as diverse as death and metamorphosis

**Related research article** Ericson CF, Eisenstein F, Medeiros JM, Malter KE, Cavalcanti GS, Zeller RW, Newman DK, Pilhofer M, Shikuma NJ. 2019. A contractile injection system stimulates tubeworm metamorphosis by translocating a proteinaceous effector. *eLife* **8**:e46845. doi: 10.7554/eLife.46845

**Related research article** Vlisidou I, Hapeshi A, Healey JRJ, Smart K, Yang G, Waterfield NR. 2019. The PVC element of *Photorhabdus asymbiotica* virulence cassettes deliver protein effectors directly into target eukaryotic cells. *eLife* **8**:e46259. doi: 10.7554/eLife.46259

Ever since the emergence of life on earth all types of organisms have had to evolve clever strategies to cope with each other and with their changing environment. In bacteria and other micro-organisms survival often involves deploying toxins to eliminate other microbes. This can be done by using a molecular machine called the type VI secretion system (T6SS) to inject toxins into competitors (Ho et al., 2014). However, some bacteria take a different approach, releasing injection systems into the extracellular medium, from where they bind to the target organism and inject toxins or other proteins (Böck et al., 2017). Now, in eLife, two independent groups report new insights into these extracellular injection systems, which are less well understood than the T6SS.

It has been known for some time that bacteria can trigger the metamorphosis of larvae into adults in various animal species (Yang et al., 2013): for example, the marine bacterium *Pseudoalteromonas luteoviolacea* releases extracellular injection systems called MACs (short for metamorphosis-associated contractile structures) that trigger the metamorphosis of the tubeworm *Hydroides elegans* (Shikuma et al., 2014). Whereas the T6SS consists of a contractile sheath wrapped around an inner tube, a MAC array consists of about 100 contractile sheaths packed together. Upon contraction of the sheath, whatever is inside is injected into the target.

In the first paper Nicholas Shikuma (UC San Diego), Martin Pilhofer (ETH Zurich) and co-workers – including Charles Ericson and Fabian Eisenstein as joint first authors – used cryo-electron tomography to directly visualise a density in the inner tubes of a MAC array, which they propose is the toxin that is injected into targets (Ericson et al., 2019). Further experiments show that the toxin is a protein called Mif1: first, the density is not seen in bacteria in which the gene for Mif1 has been deleted; second, Mif1 co-purifies with the MAC arrays when they are prepared from the bacterial supernatant; third, electroporation of purified Mif1 into the tubeworm larvae induces metamorphosis. Together these experiments show that metamorphosis exclusively relies on Mif1 and that the MAC array is the delivery device. Other recent work by Shikuma, Pilhofer and co-workers has shown that MAC arrays can also deliver a nuclease called Pne1 that is cytotoxic to a range of eukaryotic cells (Rocchi et al., 2019).

The cryotomography images produced by Ericson et al. are notable as the cargos can be seen inside the contractile sheaths (Figure 1). Previously researchers had co-purified the antibacterial toxin Tse2 and the hexameric rings that make up the inner tube of T6SS, and had seen the toxin inside the individual rings (Silverman et al., 2013), but the latest work is the first time a toxin has been seen inside an intact injection system.

**Figure 1.**
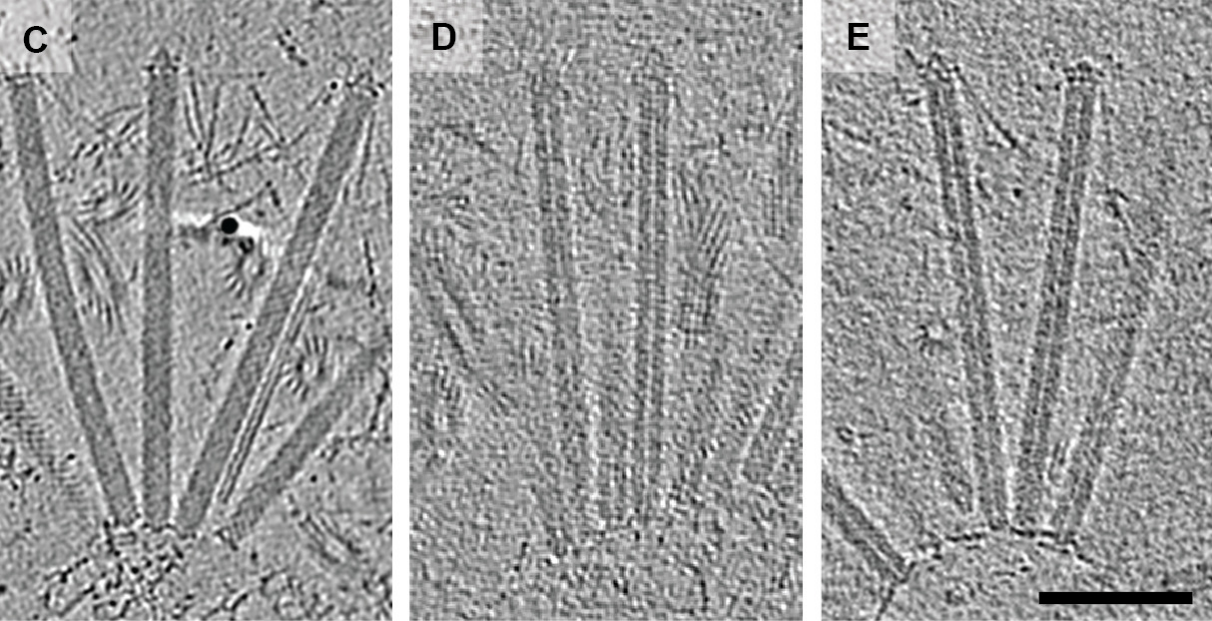
Using cryo-electron tomography to look at MACs. Cryotomographic images of wild-type MACs (left), and two mutant MACs. It can be clearly seen that the wild-type MACs contain a cargo, whereas the mutant MACs are empty. The scale bar is 100 nanometres long.

In the second paper, Nicholas Waterfield (Warwick University), Guowei Yang (Capital Medical University) and co-workers – including Isabella Vlisidou (University Hospital of Wales) as first author – report the results of experiments on an extracellular contractile injection system called the *Photorhabdus* virulence cassette (PVC; Vlisidou et al., 2019). *Photorhabdus* is a bioluminescent bacterium with a remarkable life cycle: it establishes a symbiotic relationship with pathogenic nematodes that can penetrate into insect larvae and release *Photorhabdus;* in turn the bacteria produce multiple toxic compounds, including PVC devices, which kill the insect, thus providing the nematodes with a source of nutrients (McQuade and Stock, 2018). The PVC device is similar to the MAC array in that it is an extracellular injection system and is similar to T6SS in that it has just one contractile sheath.

Vlisidou et al. show that an effector toxin called Pnf (short for *Photorhabdus* necrosis factor) is bound to the PVC devices. Although it remains to be shown that delivery of Pnf into a target requires a functional PVC device, a clear impact on the cytoskeleton (which is a popular target for effector toxins; Lerm et al., 2000) was observed when purified Pnf protein was transfected into HeLa cells, but not when Pnf was added extracellularly. This suggests that PVC devices are needed to get the toxin inside the target.

Vlisidou et al. also show that Pnf has tranglutaminase and deamidase activity on a range of purified small GTPases, notably RhoA and Rac1, which in turn disrupts the cytoskeleton organisation through two reactions. The researchers were able to pin down this role of Pnf thanks to its homology with CNF2, a toxin released by *Yersinia pseudotuberculosis* (a bacterium that can cause fever in humans) that deamidates members of the Rho family (Hoffmann et al., 2004).

An outstanding question in the field of contractile injection systems – both intracellular systems like T6SS and extracellular systems like MAC and PVC – concerns how the toxins are delivered. Ericson et al. and Vlisidou et al. both show a direct association between the toxin and the contractile device, and the direct visualization of Mif1 within the inner tube of the MAC array by Ericson et al. demonstrates the potential of advanced microscopic techniques to elucidate mechanisms at molecular levels.

Working out the function of toxins and effector proteins also remains a challenge. Homology can help, as Vlisidou et al. showed with Pnf, but many effectors identified through genomic or secretome analysis have no characterised homologues. This is definitely the case for Mif1 and its role in metamorphosis.

And how do bacteria select the effectors and toxins to be loaded into these systems? The MAC array, for example, can deliver Mif1, Pne and other cargos – are they delivered simultaneously? This is an important question because the different cargos lead to very different outcomes (developmental support in the case of Mif1, toxicity for Pne). There is thus an increasingly complex love-hate relationship between organisms. All options remain open, allowing the environment to decide on the most beneficial outcome: basic Darwinian evolution, in other words.
